# Reduced obstacles, maximized vision (ROMV): a new technique to facilitate laryngoscopy for endotracheal intubation

**DOI:** 10.1080/03009734.2016.1246494

**Published:** 2016-11-16

**Authors:** Mohammad Kharazmi, Håkan Scheer, Pär Hallberg

**Affiliations:** a Section of Orthopaedics, Department of Surgical Sciences, Uppsala University SE-751 85 Uppsala, Sweden;; b Department of Oral and Maxillofacial Surgery, Västmanland Hospital Västerås, SE-721 89 Västerås, Sweden;; c Department of Anaesthesiology and Intensive Care, Västmanland Hospital Västerås, SE-731 30 Västerås, Sweden;; d Department of Medical Sciences, Uppsala University, SE-751 85 Uppsala, Sweden

Laryngoscopy is a procedure performed to obtain a view of the vocal folds and the glottis. It is used for tracheal intubation and also to gain access for treatment on the larynx or other parts of the upper airway. The outcome of the procedure is highly dependent on the skills of the operator. Incorrectly executed, it may jeopardize the airways. However, complications can occur even when the procedure is successful, such as traumatic injuries in the oral cavity caused by the laryngoscope blade. The incidence of dental trauma following laryngoscopy is up to 12% (1) and is often due to insufficient mouth opening. Insufficient mouth opening not only leads to a lack of space for the laryngoscope, but also impaired vision, requiring the lower jaw to be bent down to enable access to the oral cavity. Manipulation of the lower jaw combined with impaired vision can lead to an undesirable angulation of the laryngoscope, which is a main cause for trauma of the soft tissues occurring in more than half of patients undergoing laryngoscopy (2). We here describe a simple technique that can reduce obstacles and maximize vision (ROMV) during laryngoscopy. These benefits offer a means to reduce the risk of trauma-related complications and may also improve the success rate of the procedure.

**Figure 1.**
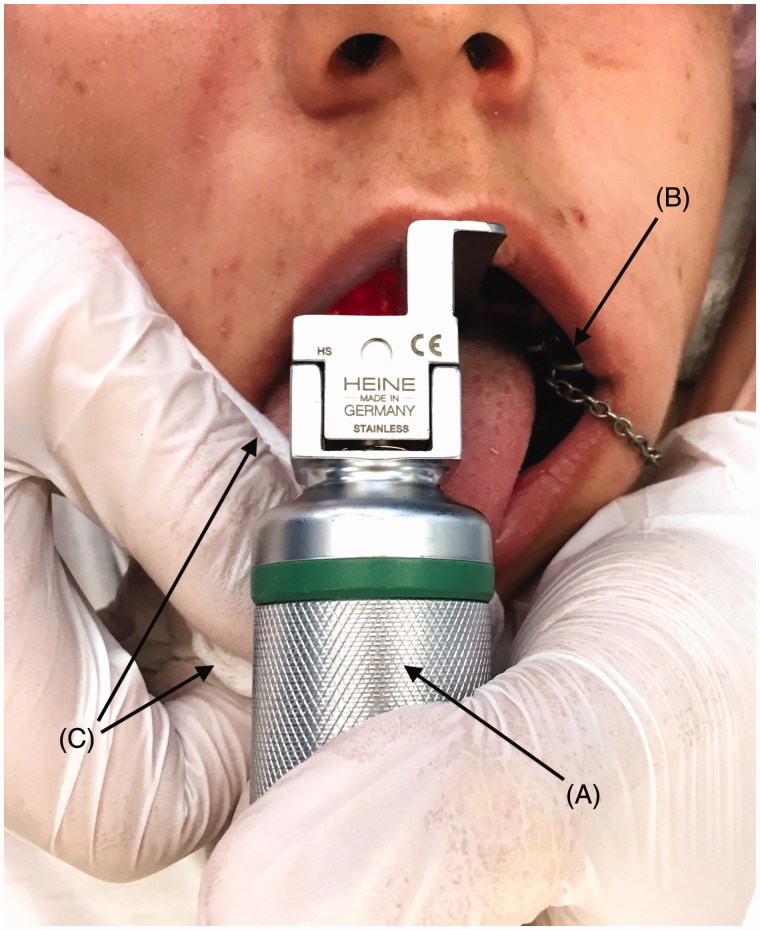
Usage of the ROMV technique during laryngoscopy. Note the wide opening of the mouth and the readily accessible area towards the posterior plate and the pharynx. A: Laryngoscope with size 3 Macintosh blade. B: Bite block applied to the left side of the mouth to maintain wide opening of the mouth, hence not requiring manipulation of the lower jaw. C: Gauze is used to grasp the tip of the tongue with the right hand and gently to pull it outwards.

Prior to laryngoscopy, a bite block is inserted on the side of the mouth between the upper and lower posterior teeth (Figure 1). The tip of the tongue is grasped with gauze with one hand and the tongue is gently pulled outwards. Next, the laryngoscope is inserted, and the tongue is released once a clear vision of the vocal cords is obtained. After successful insertion of the endotracheal tube, the bite block is removed.

The only difference between ROMV and standard laryngoscopy is the use of a bite block combined with outwards traction of the tongue. The use of a bite block offers several advantages. It increases mouth opening, improves vision into the oral cavity, and reduces the need for manipulation of the lower jaw. By gently pulling the tongue outwards, the airways are opened and visibility of the pharynx is improved. It also allows more room for the laryngoscope to slide down behind the tongue. The anterior traction achieved by a gentle pull of the tongue will also elevate the epiglottis, hence improving vision of the vocal cords. Control of the tongue also facilitates placing of the tube in such a way as to avoid unwanted pressure on the tongue, thereby decreasing the risk of tissue necrosis (3).

Traction of the tongue is a method known to anaesthesiologists for quite some time. Also, the value of using a bite block was recently described by Kishimoto et al. (4). However, the ROMV technique combines the two so as to attain maximum benefit. The technique is non-invasive, safe, and quick. Based on experience from 10 patients at our clinic we have estimated the average additional time required for a non-experienced operator to implement ROMV as compared to standard laryngoscopy to less than 15 seconds.

ROMV should be regarded as a complement to standard laryngoscopy, used by the operator in specific cases. Such cases could include patients with previous trauma to the anterior teeth, patients with an increased risk of luxation of the temporomandibular joints or an increased risk of oral trauma (prominent mandibular torus or mandibular shelves) (5), or patients with severe hyposalivation associated with risk of soft tissue trauma due to lack of natural mucosal lubrication. The ROMV technique could also provide support for non-experienced operators.

There are some limitations of the technique. The bite block can only be used on patients with stable destination or it may cause more harm than benefit. In children, a bite block may cause increased mobility of already mobile primary teeth. Also, traction of the tongue is contraindicated in patients with ankyloglossia. Hence, the mobility of the patient’s tongue needs to be evaluated prior to laryngoscopy. It should also be acknowledged that digital techniques such as videolaryngoscopy or fibre-optics provide an unparalleled increase in vision. Hence, our technique should be regarded as a complement to traditional laryngoscopy and not a replacement for digital techniques.
